# Microsatellite instability and Epstein-Barr virus combined with PD-L1 could serve as a potential strategy for predicting the prognosis and efficacy of postoperative chemotherapy in gastric cancer

**DOI:** 10.7717/peerj.11481

**Published:** 2021-05-18

**Authors:** Na Yang, Yanhua Wu, Meishan Jin, Zhifang Jia, Yueqi Wang, Donghui Cao, Lili Qin, Xueying Wang, Min Zheng, Xueyuan Cao, Jing Jiang

**Affiliations:** 1 Division of Clinical Research, First Hospital of Jilin University, Changchun, Jilin Province, China; 2 Division of Pathology, First Hospital of Jilin University, Changchun, Jilin Province, China; 3 Department of Epidemiology and Biostatistics, School of Public Health, Jilin University, Changchun, Jilin Province, China; 4 Department of Gastric and Colorectal Surgery, First Hospital of Jilin University, Changchun, Jilin Province, China

**Keywords:** Microsatellite instability, Epstein-Barr virus, PD-L1, Gastric cancer, Postoperative chemotherapy, Overall survival

## Abstract

**Background:**

Microsatellite instability (MSI) and Epstein-Barr virus (EBV)-positive molecular subtypes exhibit complex immune responses in gastric cancer (GC), and PD-L1 has emerged as a prognostic biomarker associated with the cancer immune microenvironment. This study aimed to determine the prognostic value of molecular subtypes and whether the addition of PD-L1 would accurately predict the prognosis and guide postoperative chemotherapy for GC patients.

**Methods:**

We performed molecular subtyping of tissue microarray slides from 226 GC patients who were treated with radical gastrectomy. The MSI status and PD-L1 expression were evaluated through immunohistochemistry (IHC) and EBV status through situ hybridization. Multiplex polymerase chain reaction (PCR) was also performed on 50 cases to validate the accuracy of IHC in defining MSI status. Differences in overall survival (OS) were assessed using the Kaplan-Meier method, log-rank test and Cox proportional hazards regression model.

**Results:**

Among the 226 GC patients, 52 (23.2%) patients were classified as the MSI subtype, 11 (4.9%) were EBV^+^ subtype, and 161 (71.9%) were MSS (Microsatellite stable) /EBV subtype according to TCGA analysis. Two patients were both positive for MSI and EBV infection. EBV^+^ cases showed higher PD-L1 positivity than MSI cases and MSS/EBV cases (81.8% *vs.* 50.0% *vs.* 35.4%, *P* = 0.003). Compared with the non-MSS/EBV (MSI or EBV^+^ cases) subgroup, GC patients with MSS/EBV were associated with the worst outcomes (HR = 1.610, 95% CI [1.0462.479], *P* = 0.031). MSS/EBV GCs alone could benefit from postoperative chemotherapy (HR = 0.452, 95% CI [0.2990.682], *P*<0.001), and PD-L1-positive expression could also predict a better prognosis (HR = 0.612, 95% CI [0.3890.962], *P* = 0.033) in this subgroup. Considering both chemotherapy efficacy and PD-L1 expression in the MSS/EBV subgroup, chemotherapy could improve the prognosis for PD-L1-negative MSS/EBV GCs (HR = 0.357, 95% CI [0.2170.587], *P* <0.001) but not PD-L1-positive MSS/EBV GCs.

**Conclusions:**

Molecular subtyping combined with PD-L1 expression could serve as a potential strategy to better predict prognosis and guide postoperative chemotherapy of GC patients.

## Introduction

Gastric cancer (GC) is still an important global health problem (Lyons et al., 2019) and ranks fifth in terms of incidence but third in terms of mortality, with over 1,000,000 new cases and an estimated 769,000 deaths worldwide in 2020 (Sung et al., 2021). Notably, more than 40% of the worldwide GC cases and deaths occur in China. Based on thelatest data from the National Central Cancer Registry of China (NCCRC) in 2015,GCwas the second most prevalent cancer and third leading cause of cancer-related deaths in China (Wang et al., 2019). At present, GC patients are still mainly treated with complete tumor resection, and postoperative adjuvant radiotherapy and chemotherapy or neoadjuvant chemotherapy can improve the survival time of patients with advanced GC (Tan, 2019). Surgical resection combined with chemotherapy remains the first-line treatment for advanced GC in China. Even though the GC survival rate has increased due to the improved systemic management over recent decades, the prognosis of GC patients remains poor in China, with a disappointing low 5-year relative survival rate of 35.9% (Yang etal., 2018). More importantly, a variable response to treatment has been observed based on the one-size-fits-all approach to treatment, showing that GC is a heterogeneous disease (Digklia & Wagner, 2016). Thus, there is an urgent need for novel biomarkers to precisely predict the prognosis of GCs and to achieve personalized treatment.

The Cancer Genome Atlas (TCGA) has recently proposed four comprehensive molecular subtypes for GC based on gene expression profile studies (Cancer Genome Atlas Research Network, 2014), which provide new prognostic biomarkers and potential therapeutic implications, particularly the Epstein-Barr virus (EBV)-positive subtype and microsatellite instability (MSI) subtype (Kawazoe et al., 2017). Recent studies have suggested that patients with different molecular subtypes of GC may have different prognoses, and some subtypes, such as MSI, may affect the efficacy of chemotherapy for GC (Smyth et al., 2017). However, in this regard, molecular typing alone may not be sufficient, and more markers should be considered for prognostic evaluation and choice of treatment. Previous studies indicate that the EBV and MSI subtypes may show complex reactions in the immune system, which exhibit high levels of tumor infiltrating lymphocytes (Chang et al., 2018). It indicates that the immune microenvironment of the tumor and tumor infiltration may be different in GC patients with different molecular types. PD-L1 has emerged as the most promising biomarker, which is associated with the cancer immune microenvironment (Jin et al., 2017). PD-L1 expression in tumor or tumor infiltrating immune cells are used as an adjunct diagnostic criteria for the application of PD-1/PD-L1 pathway inhibitors (Muro et al., 2016), but the prognostic value of PD-L1 is controversial in GC patients (Liu et al., 2020). In recent years, higher PD-L1 expression has been found to be closely associated with EBV-positive and MSI GCs (Lee et al., 2017; Derks et al., 2016). However, it is unclear whether the value of PD-L1 expression in predicting GC prognosis is contingent upon EBV and MSI statuses (Choi et al., 2020). Overall, given the complex immune interactions that occur within the tumor microenvironment and available evidence, the addition of PD-L1 expression based on stratification by EBV and MSI statuses would provide better prognostic insight and elucidate the therapeutic implications in GC, whereas the available reports regarding this pattern are insufficient.

In this study, we focused on the molecular subtypes stratified by EBV and MSI statuses according to the TCGA study and analyzed their prognostic value. Furthermore, we examined the level of PD-L1 expression and explored its prognostic value, as well as its role in response to postoperative chemotherapy among different molecular subtypes for GC patients.

## Materials & Methods

### Study population

A total of 226 cases were obtained from the Department of Gastric and Colorectal Surgery, First Hospital of Jilin University (Changchun, China) from February 2011 to August 2016. All included patients were diagnosed with GC pathologically, each of whom was treated with radical gastrectomy and did not receive any chemotherapy or radiotherapy prior to surgery. Patients with distant metastasis or a positive surgical margin were excluded. We retrieved the information of clinical characteristics from the electronic medical record system, which includes age, gender, tumor size, WHO classification, postoperative chemotherapy, T stage, N stage, histological grade, vascular invasion, and neural invasion. The AJCC Cancer Staging Manual (8th edition) was applied to determine the TNM stage.

### Follow-up data collection

Prospective follow-up of GC patients was conducted regularly at three months and six months after gastrectomy, as well as every year afterwards until death or being lost to follow-up. The prognosis and chemotherapy of patients were collected during the follow-up. Overall survival (OS) was calculated from surgery to death (from any cause) or the final date of follow-up (if the patients were alive) or the last date of follow-up (if the patients were lost to follow-up). Cases would not be included in the survival analysis if they were lost to follow-up at the first time of telephone interview or if they died of perioperative complications. Postoperative chemotherapy was defined as at least three cycles of chemotherapy after surgery, which mainly included the following three regimens: XELOX (oxaliplatin and capecitabine), FOLFOX-4 (5-fluorouracil, leucovorin and oxaliplatin) and other (such as oxaliplatin, capecitabine or other combinations).

### Immunohistochemistry

Immunohistochemistry (IHC) was carried out formalin-fixed, paraffin-embedded 226 GC tissues to detect the expression of four MMR proteins and PD-L1 with antibodies to MLH1 (ab92312, dilution: 1/200, Abcam, Cambridge, UK), MSH2 (ab92473, dilution: 1/200, Abcam, Cambridge, UK), MSH6 (ab92471, dilution: 1/200, Abcam, Cambridge, UK), PMS2 (ab110638, dilution: 1/200, Abcam, Cambridge, UK), and PD-L1 (E1L3N, dilution: 1/200, Cell Signaling Technology, Cambridge, UK). Among them, tissue microarray slides (MiniCore, Alphelys, France) were used for MMR proteins detection and whole sections were used for PD-L1 detection. First, the slides were baked in a 65C oven for 20min, followed by dewaxing. Antigen retrieval was performed in sodium citrate aqueous solution in 100C for one minute and forty seconds (20min and ethylenediamine tetraacetic acid for PD-L1 without high pressure). After this, the slides were washed 3 times with PBS for 5min each time. The slides were incubated with endogenous peroxidase blocker for 10min at room temperature, then washed 3 times with PBS for 5min each time. Then, the slides were incubated with primary antibody for 60min (90min for PD-L1) at room temperature, followed by secondary antibody for 15min at room temperature and washed 3 times with PBS for 5min each time. 3, 3-diaminobenzidine tetrahydrochloride (DAB) H_2_O_2_ was added for 2min. Counterstaining was done with hematoxylin.

### MSI analysis

Positive expression of each MMR protein in IHC was defined as the presence of nuclear staining of tumor cells, regardless of the proportion or intensity (Smyth et al., 2017). Stromal cells of tumor tissue served as the internal negative control. Two pathologists judged the immunohistochemical staining for the expression of four MMR proteins in a blinded manner. MSS was determined with expressions of all MMR proteins, and MSI was determined with at least one MMR protein showing a complete loss of nuclear reactivity (Smyth et al., 2017). For cases with any MMR protein loss, the experiments were repeated on whole sections, and the results were reanalyzed by two pathologists for final confirmation.

To validate the accuracy of the IHC method, we performed multiplex polymerase chain reaction (PCR) on 50 cases randomly selected from the MSI (8 cases) and MSS (42 cases) groups. Genomic DNA was extracted from tumor tissues and matchedto adjacenttissues using the tissue DNA isolation mini kit (Vazyme Biotech Co., Ltd., Beijing, China) according to the manufacturers instructions. Multiplex PCR was performed with six mononucleotide repeat markers (BAT-25, BAT-26, NR-21, NR-24, NR-27, and MONO-27) using a multiplex PCR-capillary electrophoresis MSI detection kit (Sinomdgene Co. Ltd., Beijing, China). Amplified PCR products were separated, and their sizes were evaluated by capillary electrophoresis on an Applied Biosystems 3130 or 3500 Dx genetic analyzer (Los Angeles, CA, USA). GeneMapper software (Applied Biosystems) was used to analyze allelic sizes. By comparing the allelic position of the microsatellite markers in tumor and matched normal tissues, cases with instability at two or more microsatellite markers were classified as MSI-high (MSI-H), whereas cases with instability at 1 microsatellite marker and those without instability were classified as MSI-low (MSI-L) and microsatellite stable (MSS), respectively (Goel et al., 2010). Both MSI-L and MSS tumors were combined as MSS in our analysis, according to previous studies (Chao et al., 2019).

### EBV insitu hybridization

The evaluation of EBV infections were confirmed via DNA in situ hybridization (ISH-5021, ZSGB-BIO, China) using tissue microarrays (MiniCore, Alphelys, France). Tumors with brown nuclear staining were considered as EBV-positive (Pereira et al., 2018).

### PD-L1 expression analysis

Cell membrane and cytoplasm staining were performed to detect PD-L1 expression in tumor cells (TCs). The IRS system was used to assess the staining intensity and percentages of TCs (Wu et al., 2017). Staining intensity and expression prevalence were graded using a 4-point scale as follows: 0 (no immunostaining; <5% expression), 1 (weak staining; 5 to 19% expression), 2 (moderate staining; 20 to 49% expression), or 3 (strong staining; 50% expression), and the percentages of PD-L1-positive TCs were recorded as the following 4 categories: 0 (<5% expression), 1 (5 to 19% expression), 2 (20 to 49% expression) and 3 ( 50% expression). In the IRS system, specimens were scored on the basis of the intensity and percentage scores, ranging from 0 to 6. A total score of more than 2 indicated a PD-L1-positive status.

### Statistical analysis

The data were analyzed by SPSS version 21.0 for Windows (SPSS Inc., Chicago, IL, USA). The difference of PD-L1 positivity between the three molecular subgroups was tested using Pearson **^2^ or Fishers exact test when appropriate. Differences in OS by molecular features and other characteristics were assessed using the KaplanMeier method, log-rank test and Cox proportional hazards regression model. Hazard ratios (HRs) and their 95% confidence intervals (CIs) for OS were calculated. All tests were two-sided, and *P* values < 0.05 were considered to be statistically significant.

### Ethics approval

This research was carried out in accordance with the Declaration of Helsinki and was approved by the Human Ethics Review Committee of the First Hospital of Jilin University (Project identification code: 2017-090).

### Consent to participate

All participants signed the written informed consents before sample and information collection.

## Results

### Patients characteristics

**Figure 1:**
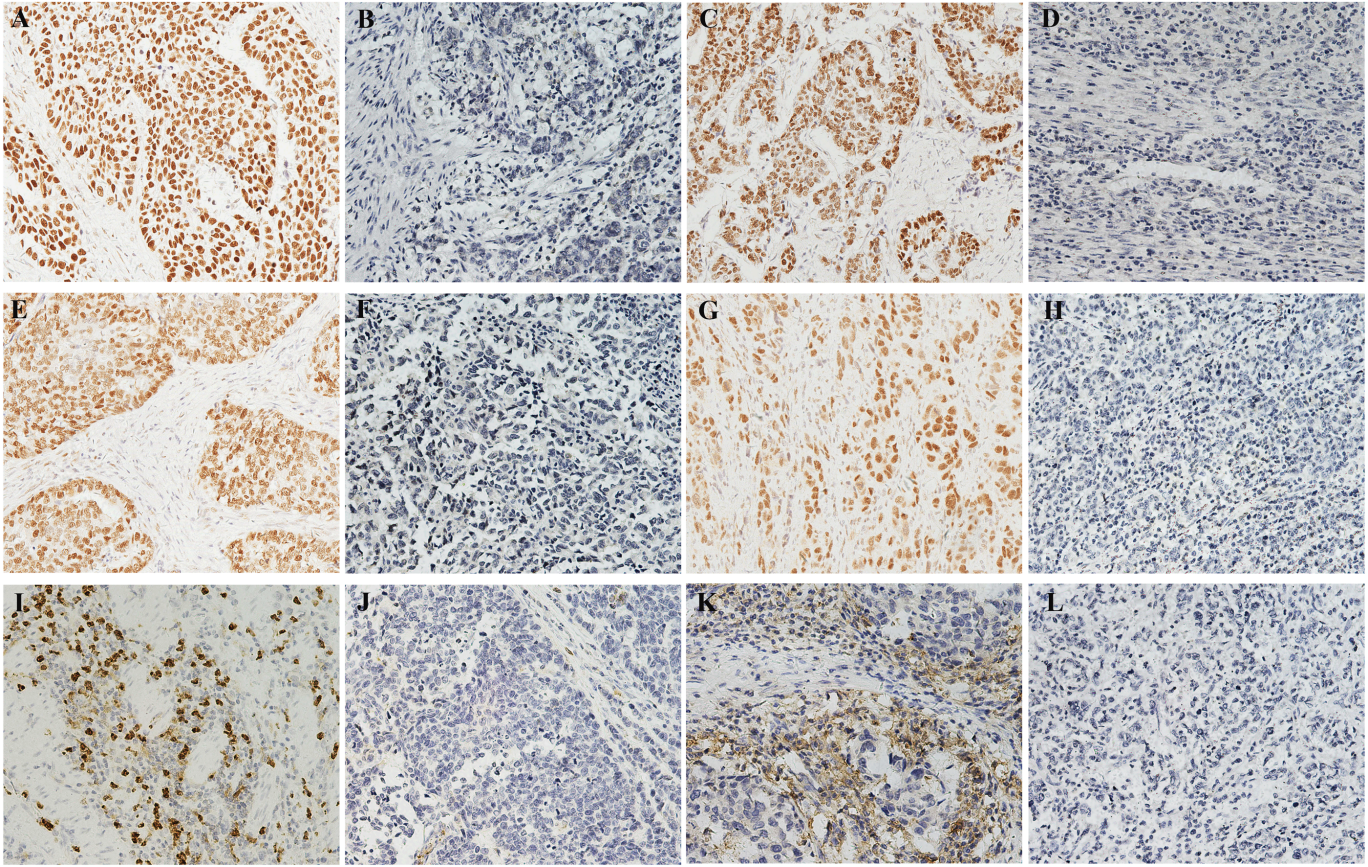
Molecular features of tumor issue. (AH) Immunohistochemistry for four MMR proteins in the nucleus of tumor cells: (A) Positive MLH1 expression. (B) The loss of the expression of MLH1. (C) Positive MSH2 expression. (D) The loss of the expression of MSH2. (E) Positive MSH6 expression. (F) The loss of the expression of MSH6. (G) Positive PMS2 expression. (H) The loss of the expression of PMS2. (I) EBV-positive case with strong brown nuclear ISH staining. (J) Negative detection of EBV in tumor cells. (K) PD-L1 expression in tumor cells (both in cell membrane and cytoplasm staining). (L) Negative detection of EBV in tumor cells. Magnification: 200.

Median follow-up was 39.7 months (range 1.086.7). The baseline characteristics of the 226 GC patients finally enrolled are shown in Table 1. Postoperative chemotherapy was administered to 101 (44.7%) patients. Of note, the majority of patients were in stage III (70.4%) in the entire cohort. The expressions of representative molecular features in GC tissue are shown in Figure1. MSI status was determined in all 226 cases by IHC. Overall, 172 (76.1%) patients exhibited MSS and 54 (23.9%) patients exhibited MSI (Table 1). The positive rates of MLH1, MSH2, MSH6, and PMS2 protein expression were 85.8%, 96.0%, 86.7%, and 85.8%, respectively (Figs. 1A1H). It has become a consensus that immunohistochemistry can be a useful and reliable method in the detection of MSI status in GC compared with microsatellite marker analysis, which could achieve high concordance (91.1%) (Bae et al., 2015). In the current study, of the total 50 cases, 6 cases showed a discrepancy between IHC and PCR results, including 2 cases of MSI-H and 4 cases of MSS/MSI-L, determined on the basis of PCR-based analysis, showing a similar high concordance (88%, Table S1), which is reliable enough for the determination of the MSI status of GCs. Among all patients assessed by ISH, 13 (5.8%) patients were positive for EBV infection (Table 1 and Fig. 1I), and 93 patients (41.2%) were positive for PD-L1 expression in TCs (Table 1 and Fig. 1K). Examples of PD-L1 graded staining in Fig. S1 and the MSI-H and MSS cases based on PCR analysis are shown in Fig. S2.

**Table 1:** Baseline characteristics of enrolled 226 gastric cancer patients.

**Characteristics**		**N (%)**
Gender	Male	172(76.1)
	Female	54(23.9)
Age(years)	<60	92(40.7)
	60	134(59.3)
Tumor size(cm)	<5	67(29.6)
	5	159(70.4)
WHO classification	Tubular adenocarcinoma	165(73.0)
	Mucoidadenocarcinoma	28(12.4)
	Signet ring cell	30(13.3)
	Others	3(1.3)
Histological grade	Low grade	54(23.9)
	High grade	172(76.1)
Vascular invasion	Negative	45(19.9)
	Positive	181(80.1)
Neural invasion	Negative	67(29.6)
	Positive	159(70.4)
CTx	Yes	101(44.7)
	No	125(55.3)
TNM stage	I/II	67(29.6)
	III	159(70.4)
Depth of invasion	T1/T2	9(4.0)
	T3/T4	217(96.0)
Lymph metastasis	N0	38(16.8)
	N1/N2/N3	188(83.2)
MSI status	MSI	54(23.9)
	MSS	172(76.1)
EBV	Positive	13(5.8)
	Negative	213(94.2)
PD-L1	Positive	93(41.2)
	Negative	133(58.8)

**Notes.**

MSI Microsatellite instability MSS Microsatellite stable EBV Epstein-Barr Virus PD-L1 Programmed cell death ligand 1 CTx Chemotherapy

### Molecular subgroups classified by MSI and EBV statuses

In the present study, we defined EBV-positive and MSI subgroups separately according to TCGA classification (Cancer Genome Atlas Research Network, 2014). Patients with MSI status were assigned to the MSI subgroup (*n*=52, 23.2%), patients with EBV infection were assigned to the EBV^+^ subgroup (*n*=11, 4.9%), and patients without MSI and EBV infection were assigned to the MSS/EBV subgroup (*n*=161, 71.9%). It is worth noting that 2 patients were both positive for MSI and EBV infection, which were not classified in the subsequent subgroup analysis.

### MSS/EBV subgroup was associated with poor prognosis in GC

**Figure 2:**
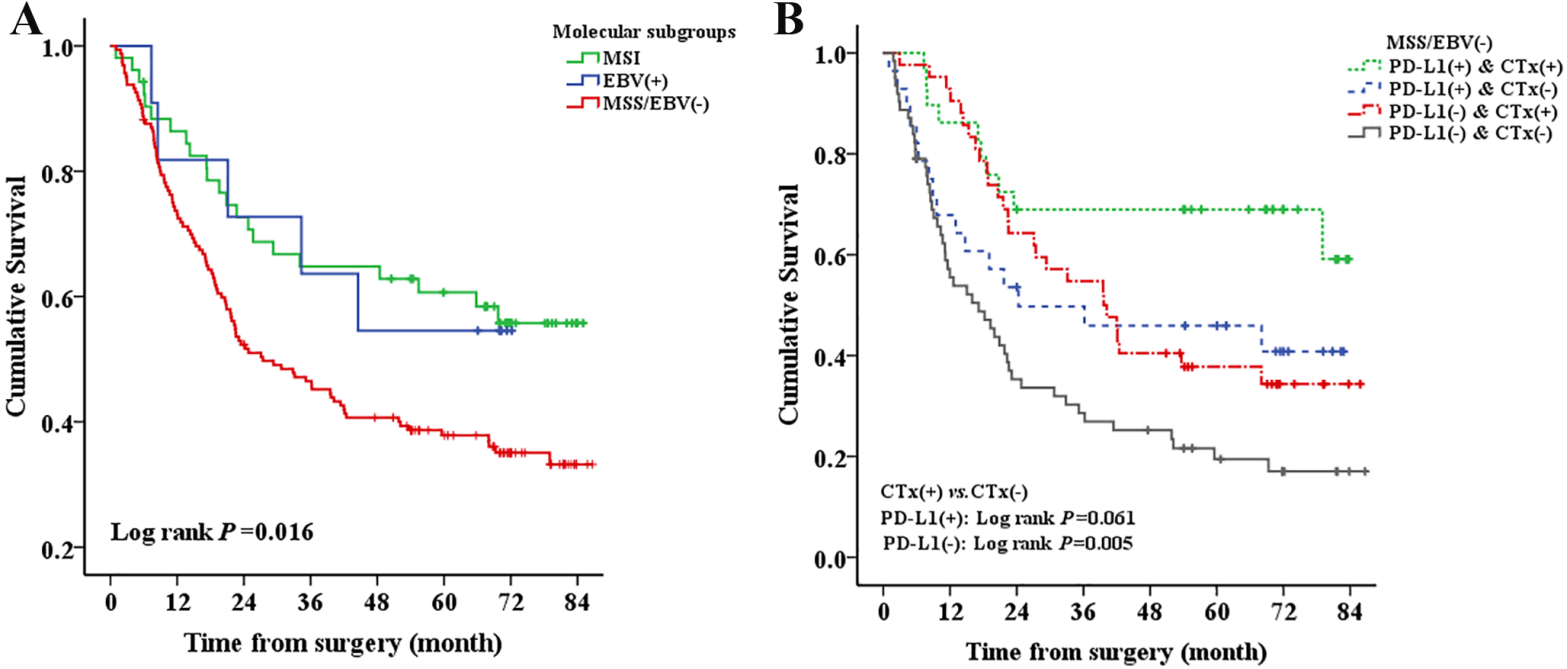
KaplanMeier estimates of overall survival (OS) of gastric cancer patients according to (A) three molecular subgroups (B) Chemotherapy in MSS/EBV/PD-L1^+^ and MSS/EBV/PD-L1 subgroup. MSI, Microsatellite instability; MSS, Microsatellite stable; EBV, Epstein-Barr Virus; PD-L1, Programmed cell death ligand 1; CTx, Chemotherapy.

Log-rank test investigated that MSS/EBV GCs showed the worst OS compared to MSI and EBV^+^ GCs (*P*=0.016, Fig. 2A). The associations between other clinical characteristics and GC survival are shown in Table S2. Results of multivariate Cox regression also showed that patients with MSS/EBV appeared to have the worst outcomes (HR = 1.547, 95% CI [0.9652.481], *P*=0.077, Table 2) compared to those with MSI. When MSI and EBV^+^ cases were combined as a reference group (non-MSS/EBV subgroup), multivariate analysis revealed that GCs with MSS/EBVhad poorer OS (HR = 1.610, 95% CI [1.0462.479], *P*=0.031, Table S3). Also, PD-L1 expression tended to correlate with improved survival, but multivariate analysis showed marginal significance (HR = 0.684, 95% CI [0.4581.021], *P*=0.063, Table 2).

**Table 2:** Multivariate analyses of risk factors affecting overall survival (OS) in 224 gastric cancer patients.

Characteristics		HR	95% CI	*P*
Molecular subtypes	MSI (*n*=52)	1.00		0.077
	EBV^+^ (*n*=11)	0.755	0.2752.076	
	MSS/EBV (*n*=161)	1.547	0.9652.481	
PD-L1	Positive	1.00		0.063
	Negative	0.684	0.4581.021	
TNM stage	I/II	1.00		0.009
	III	2.117	1.2083.709	
Vascular invasion	Negative	1.00		0.020
	Positive	2.216	1.1354.330	
CTx	No	1.00		<0.001
	Yes	0.477	0.3310.686	

**Notes.**

MSI Microsatellite instability MSS Microsatellite stable EBV Epstein-Barr virus PD-L1 Programmed cell death ligand 1 CTx Chemotherapy HR Hazard ratio CI Confidence interval

95% CI and *P* values were calculated with multivariate Cox regression, adjusting for the variables that *P*<0.100 from the univariate analysis, such as PD-L1, WHO classification, histological grade, vascular invasion, neural invasion, postoperative chemotherapy and TNM stage.

### MSS/EBV subgroup could benefit from postoperative chemotherapy

To determine the effect of postoperative chemotherapy in the MSS/EBV subgroup, which tended to have the worst clinical outcomes, we analyzed the association of chemotherapy and GC survival stratified for different molecular subgroups. The results showed that postoperative chemotherapy can improve the OS of the MSS/EBV subgroup (log-rank *P* <0.001, Fig. S3C) but showed less benefit when compared with the MSI (log-rank *P*=0.327, Fig. S3B) and EBV^+^ subgroups (log-rank *P*=0.355, Fig. S3A). Besides, further multivariate analysis in MSS/EBV subgroup showed that postoperative chemotherapy could improve OS of the MSS/EBV GCs (HR = 0.452, 95% CI [0.2990.682], *P*<0.001, Table 3)

**Table 3:** Multivariate analyses of risk factors affecting overall survival (OS) in MSS/EBV subgroup (*N*=161).

Characteristics		HR	95% CI	*P*
PD-L1	Negative	1.00		0.033
	Positive	0.612	0.3890.962	
Histological grade	Low grade	1.00		0.024
	High grade	1.834	1.0833.107	
TNM stage	I/II	1.00		<0.001
	III	3.342	1.7986.212	
CTx	No	1.00		<0.001
	Yes	0.452	0.2990.682	

**Notes.**

MSI microsatellite instability MSS microsatellite stable EBV Epstein-Barr Virus PD-L1 programmed cell death ligand 1 CTx Chemotherapy HR Hazard ratio CI Confidence interval

95% CI and *P* values were calculated with multivariate Cox regression, adjusting for the variables that *P*<0.100 from the univariate analysis, such as WHO classification, histological grade, vascular invasion, neural invasion, postoperative chemotherapy and TNM stage.

### PD-L1 expression could predict the prognosis of GC patients with MSS/EBV subtype

PD-L1 positivity was associated with the EBV^+^ cases. EBV^+^ cases manifested more PD-L1^+^ tumors compared with the MSI and MSS/EBV cases (81.8% *vs.* 50.0% *vs.* 35.4%, *P*=0.001).

Then, we evaluated the prognostic value of PD-L1 expression in different subgroups to accurately assess the prognosis of GC patients with adverse molecular typing. We found in the MSS/EBV subgroup, which tended to show the worst clinical outcomes, that PD-L1-positive GCs were found to be related to a better prognosis compared to those who were PD-L1-negative (*P*<0.001, Fig. S3F). In contrast, there was no significant survival difference between patients who were PD-L1-positive and those without PD-L1 expression for the MSI *(P* = 0.656, Fig. S3E) and EBV^+^ (*P*=0.765, Fig. S3D) subgroups. Moreover, further multivariate analysis showed that PD-L1-positive could serve as an independent predictive factor for better OS in the MSS/EBV subgroup (HR = 0.612, 95% CI [0.3890.962], *P*=0.033, Table 3).

### PD-L1 expression could predict the response to chemotherapy of GC patients with MSS/EBV subtype

Because both PD-L1 and chemotherapy can predict better OS in the MSS/EBV subgroup, we explored the association of PD-L1 expression with the efficacy of chemotherapy in the MSS/EBV subgroup. As shown in Fig. 2B, the log rank test showed that postoperative chemotherapy can improve OS of the MSS/EBV/PD-L1^+^ subgroup (log-rank *P*=0.005), while showed less benefit for the MSS/EBV/PD-L1 subgroup and the result was not statistically significant (log-rank *P*=0.061). Further multivariate analysis indicated that chemotherapy was associated with an improved prognosis for PD-L1-negative MSS/EBV GCs (HR = 0.357, 95% CI [0.2170.587], *P* <0.001; Table S4), while it was not a significant prognostic factor for PD-L1-positive MSS/EBV GCs (HR = 0.499, 95% CI [0.2231.117], *P*=0.090; Table S4).

## Discussion

Recently, more evidence has been emerged about the potential prognostic and therapeutic implications of molecular subtypes, particularly through the stratification according to EBV and MSI status. According to The Cancer Genome Atlas (TCGA) study and recent clinical trials, EBV and MSI GC subgroups exhibited high expression of PD-L1 and could benefit from therapy with PD-1/PD-L1 antibodies. However, very few studies employed combinatorial analysis of MSI, EBV-infection and PD-L1 to evaluate the prognosis and chemotherapy efficacy of gastric cancer. As postoperative chemotherapy is still the first-line treatment for GC patients, so we try to combine conventional chemotherapy with molecular subtyping and PD-L1 expression, which may help to select the patients who would benefit the most from chemotherapy and provide evidence for individualized treatment of patients with GC. In the present study, we observed that GC patients with MSS/EBV were associated with the worst prognosis compared with those with MSI and EBV^+^. Beyond that, we found that PD-L1-positive expression can predict better OS in the MSS/EBV subgroup. At the same time, MSS/EBV GCs can also benefit more significantly from chemotherapy after surgery. Nevertheless, MSS/EBV GCs with PD-L1^+^ cannot benefit from postoperative chemotherapy.

Among the 226 GC patients in our study, 54 (23.9%) patients were defined as having MSI by IHC, which was similar to that reported in TCGA cohort (22%) (Cancer Genome Atlas Research Network. 2014) and in ACRG Korean cohort (23%) (Angell et al., 2019). The incidence of EBV-associated GC ranged from 418% among different regions (Pikua et al., 2020), our result (4.9%) is identical to the data of a recent Korean study (25/569, 4.4%) (Cho, Kang & Kim, 2019) and Taiwan study (65/1248, 5.2%) (Huang et al., 2019) that used the same ISH method. A review of PD-L1 expression in gastric cancer (Vrna et al., 2018) showed that the percentage of PD-L1^+^ in the TCs ranges from 9% to 49.1% (most of the trials were performed in Asian populations), similar to our results (41.2% in TCs). Previous studies have demonstrated a mutually exclusive relationship between GCs that were EBV-positive and deemed to have MSI (Choi et al., 2020). However, two cases representing both MSI and EBV-positive were observed in a recent study (2/287, 0.70%) (Pereira et al., 2018) and in our study (2/226, 0.88%). Previous studies have demonstrated that MSI and EBV^+^ subtypes were separately associated with better prognoses (Sohn et al., 2017), and our result is in line with the results from Pereira et al. (2018) and De Rosa etal. (2018) that suggested MSS/EBV GCs showed the worst OS compared with MSI and EBV^+^ GCs. The potential mechanism underlying better survival of these two GC subtypes is that intense inflammatory infiltration observed in EBV-positive and MSI GCs may play an important role in antitumor effects. CD8^+^ cytotoxic T cells were found to be more prevalent in these two subgroups based on the stimulation of many neo-antigens (existing in MSI and EBV^+^ tumors), which can promote the elimination of TCs (Choi et al., 2020).

As a standard regimen, postoperative chemotherapy has been recommended for patients with stage II or III GC. Nevertheless, its application to all stage II or III GC patients is unnecessary and may even be detrimental to some patients (Jiang et al., 2017). Among these, GCs with MSI may experience a detrimental effect from chemotherapy (especially for 5-FU based treatment), and this finding has been supported by several studies (Kim et al., 2015). Our study yielded a similar result, namely no significant benefit from postoperative chemotherapy (5-FU-based) was observed for MSI and EBV^+^ GCs. In this regard, cancer immunotherapy might offer a promising new option and has been demonstrated effective in MSI and EBV^+^ GCs. A recent phase II trial described the high overall response rates in MSI-H and EBV-positive GCs with pembrolizumab treatment in metastatic GCs (ORR: 85.7% for MSI-H and 100.0% for EBV-positive) (Kim et al., 2018). A possible explanation is that both MSI and EBV^+^ GCs are characterized by elevated mutation rates and high expression of PD-L1 and PD-L2, which can be used as possible targets for immunotherapy (Sohn et al., 2017), especially PD-1/PD-L1 directed therapy (Le et al., 2017). On the other hand, our results indicated that MSS/EBV GCs, which was associated with a poorer prognosis, showed improved OS with the administration of chemotherapy after surgery.

In our study, we observed that both EBV-positive and MSI GCs showed a relatively higher proportion of PD-L1^+^ expression than MSS/EBV GCs. This result is consistent with reports in previous studies (Angell et al., 2019), which can be explained by observed amplification of the PD-L1 gene in EBV-positive GCs by TCGA study (Cancer Genome Atlas Research Network. 2014) and adaptive immune resistance in the tumor microenvironment. Intratumor lymphocytes can release interferon- to induce the upregulation of PD-L1 in TCs (Pereira et al., 2018). However, the positive expression rate of PD-L1 in MSS/EBV TCs varies greatly in different studies though there is few relevant studies, which could be attributed to the various method of PD-L1 expression determination and cut-off value of calling positive status used among different studies. In the study of De Rosa etal. (2018), only 3% PD-L1^+^ cases were found in MSS/EBV subgroup and a case was considered positive if membrane immunostaining was present in at least 5% of tumor cells, independently of the intensity. Choi et al. (2020) found 14% PD-L1-positive cases in MSS/EBV when considering both staining intensity and percentage of stained tumor cells, and any membranous staining was regarded as positive expression. In the present study, a higher proportion of PD-L1^+^ cases in MSS/EBV group (35%) are reported when scoring PD-L1 expression on the basis of both the intensity and percentage of PD-L1-positive TCs and considering both cell membrane and cytoplasm staining. We combined molecular subtypes with PD-L1 expression and found that PD-L1^+^ can predict a better OS in MSS/EBV subgroup but not in the other two subgroups. In contrast to our results, De Rosa etal. (2018) reported that PD-L1^+^ cannot predict the prognosis for GC patients with MSS/EBV; however, the reliability of their result is doubtful due to a very limited number of cases with PD-L1^+^ (*n*=2) in the MSS/EBV subgroup (*n*=68). PD-L1 expression in TCs might be attributed to the response to endogenous host immunity (Kim et al., 2016). For the MSS/EBV subtype with a lower immune infiltration, the expression of PD-L1 might suggest the activation of the immune response in the tumor microenvironment, indicating a better prognosis.

Although MSS/EBV GCs can benefit from chemotherapy, it is still unclear whether there are new markers, especially PD-L1, that could subgroup them and better guide the selection of chemotherapy or immunotherapy. In the current study, chemotherapy could improve the prognosis for PD-L1-negative MSS/EBV GCs but not for PD-L1-positive MSS/EBV GCs. One possible explanation is that the survival benefit of PD-L1^+^ in this subtype could be attenuated by the immunosuppression caused by chemotherapy toxicity (Choi etal., 2019). Thus, for MSS/EBV GCs with PD-L1, with an obvious benefit from chemotherapy, chemotherapy after surgery should be recommend for GCs of this subgroup. Even though MSS/EBV GCs with PD-L1^+^ exhibit better OS, we cannot give up evaluating the potential use of immune checkpoint inhibitors targeted at PD-L1 for this distinct subgroup of GCs considering the low efficacy of chemotherapy.

Therefore, we suggest that stratification by MSI and EBV statuses combined with PD-L1 expression should be used as a potential strategy to predict prognosis and identify GC patients who are most likely to respond to chemotherapy after surgery. This predictive strategy has the potential to guide patient subtype-oriented therapeutic decisions and avoid treating a certain proportion of resectable GC patients with unnecessary treatment.

Some limitations in the present study should be addressed. Firstly, only 13 patients were identified as EBV^+^ due to the low incidence of EBV-associated GC among Chinese people, and this could lead to a low level of statistical efficacy in EBV subgroup analysis. Further study with more patients is needed to verify our results. Secondly, the median follow-up time (39.7 months) was relatively short in our study. Clinical outcomes were not observed in 42% patients, and longer follow-up was needed. With the development of clinical practice, there have been PD-L1 test kit (PD-L1 IHC 22C3 pharmDx) approved by FDA as a companion diagnostic assay for the use of pembrolizumab in GC clinical treatment (Fashoyin-Aje et al., 2019). However, due to our earlier research, we used PD-L1 antibodies for scientific research, and the consistency between them needs further study.

## Conclusions

In summary, we observed that MSS/EBV GCs were associated with the worst OS compared to that of GC patients with MSI and EBV^+^ subtypes. PD-L1 expression was associated with the MSI and EBV^+^ subtypes. MSS/EBV GCs could also gain a survival benefit from postoperative chemotherapy. On the other hand, PD-L1 positivity could predict a better prognosis for MSS/EBV GCs, whereas MSS/EBV GCs with PD-L1^+^ cannot gain survival benefit from chemotherapy. Thus, molecular typing plus PD-L1 expression could improve prognostic prediction for GC patients and help choose more effective treatments for specific subsets of GCs.

##  Supplemental Information

10.7717/peerj.11481/supp-1 Supplemental Information 1 Comparison of consistency between IHC and PCR analysis of MSI (*N*=50) MSI, Microsatellite instability; MSI-L, Microsatellite instability-low; MSI-H, Microsatellite instability-high; MSS, microsatellite stable.

10.7717/peerj.11481/supp-2 Supplemental Information 2 Univariate analysis of molecular features, clinicopathological characteristics and survival of 224 gastric cancer patients MSI, Microsatellite instability; MSS, microsatellite stable; EBV: Epstein-Barr Virus; PD-L1, programmed cell death ligand 1; CTx, Chemotherapy. ^*a*^For these characteristics, less than half of patients were died, so mean overall survival (OS) time was presented when median OS could not be calculated.

10.7717/peerj.11481/supp-3 Supplemental Information 3 Multivariate analyses of risk factors affecting overall survival (OS) in 224 gastric cancer patients MSS: microsatellite stable; EBV, Epstein-Barr Virus; PD-L1, programmed cell death ligand 1; CTx, Chemotherapy. HR: Hazard ratio; CI, Confidence interval. ^*a*^95% CI was calculated with multivariate Cox regression, adjusting for the variables that *P*<0.10 from the univariate analysis, such as WHO classification, histological grade, vascular invasion, neural invasion, postoperative chemotherapy and TNM stage.

10.7717/peerj.11481/supp-4 Supplemental Information 4 Multivariate analyses of risk factors affecting overall survival (OS) in MSS/EBV subgroup stratified for different PD-L1 expression (*N*=161) MSS, microsatellite stable; EBV, Epstein-Barr Virus; PD-L1, programmed cell death ligand 1; CTx, Chemotherapy; HR, Hazard ratio; CI, Confidence interval. ^*a*^95% CI was calculated with multivariate Cox regression, adjusting for the variables that *P*<0.10 from the univariate analysis, such as WHO classification, histological grade, vascular invasion, neural invasion, postoperative chemotherapy and TNM stage.

10.7717/peerj.11481/supp-5 Supplemental Information 5 PD-L1 graded staining intensity from 0-4 in tumor cell membrane and cytoplasm (A) Grade 0, no immunostaining. (B) Grade 1, weak staining. (C) Grade 2, moderate staining. (D) Grade 3, strong staining.

10.7717/peerj.11481/supp-6 Supplemental Information 6 PCR analysis of MSI status (A) An example of MSI-H case exhibiting alteration of allelic position of all six microsatellite markers. (B) A MSS case without instability at any microsatellite markers.

10.7717/peerj.11481/supp-7 Supplemental Information 7 KaplanMeier estimates of overall survival (OS) of gastric cancer patients according to (A) CTx in EBV^+^ subgroup (B) CTx in MSI subgroup (C) CTx in MSS/EBV subgroup (D) PD-L1 expression in EBV^+^ subgroup (E) PD-L1 expression in MSI subgroup (F) PD-L1 expression in MSS/EBV subgroup. MSI, Microsatellite instability; MSS, Microsatellite stable; EBV, Epstein - Barr virus, PD-L1, Programmed cell death ligand 1; CTx, Chemotherapy.

10.7717/peerj.11481/supp-8 Supplemental Information 8 Raw data

## Additional Information and Declarations

### Data Availability

The following information was supplied regarding data availability: The raw measurements are available in the Supplemental File.
