# Supplementary material for: Microsatellite instability and Epstein-Barr virus combined with PD-L1 could serve as a potential strategy for predicting the prognosis and efficacy of postoperative chemotherapy in gastric cancer
Source: PeerJ. 2021 May 18;9:e11481. doi: 10.7717/peerj.11481 (PMC8139270; doi:10.7717/peerj.11481)
Supplement: Supplemental Information 1 — MSI, Microsatellite instability; MSI-L, Microsatellite instability-low; MSI-H, Microsatellite instability-high; MSS, microsatellite stable. [file peerj-09-11481-s001.docx]

**Table S1:**

**Comparison of consistency between IHC and PCR analysis of MSI (N=50)**

|  | | PCR | | total |
| --- | --- | --- | --- | --- |
|  |  | MSS/MSI-L | MSI-H |  |
| IHC | MSS | 38 | 2 | 40 |
|  | MSI | 4 | 6 | 10 |
| total | | 42 | 8 | 50 |

MSI: Microsatellite instability; MSI-L: Microsatellite instability-low; MSI-H: Microsatellite instability-high; MSS: microsatellite stable.
