# Supplementary material for: Microsatellite instability and Epstein-Barr virus combined with PD-L1 could serve as a potential strategy for predicting the prognosis and efficacy of postoperative chemotherapy in gastric cancer
Source: PeerJ. 2021 May 18;9:e11481. doi: 10.7717/peerj.11481 (PMC8139270; doi:10.7717/peerj.11481)
Supplement: Supplemental Information 2 — MSI, Microsatellite instability; MSS, microsatellite stable; EBV: Epstein-Barr Virus; PD-L1, programmed cell death ligand 1; CTx, Chemotherapy. aFor these characteristics, less than half of patients were died, so mean overall survival (OS) time was presented when median OS could not be calculated. [file peerj-09-11481-s002.docx]

**Table S2:**

**Univariate analysis of molecular features, clinicopathological characteristics and survival of 224 gastric cancer patients**

| Characteristics | Patient  (N) | Death  N (%) | Median OS  (months) | log-rank *P* |
| --- | --- | --- | --- | --- |
| Gender |  |  |  | 0.536 |
| Male | 171 | 97(56.7) | 42.1 |  |
| Female | 53 | 32(60.4) | 33.1 |  |
| Age(years) |  |  |  | 0.722 |
| <60 | 90 | 52(57.8) | 40.2 |  |
| ≥60 | 134 | 77(57.5) | 36.2 |  |
| Tumor size(cm) |  |  |  | 0.112 |
| <5 | 66 | 34(51.5) | 55.4 |  |
| ≥5 | 158 | 95(60.1) | 29.3 |  |
| WHO classification |  |  |  | 0.015 |
| Tubular adenocarcinoma | 164 | 90(54.9) | 53.6 |  |
| Mucinous adenocarcinoma | 27 | 21(77.8) | 22.3 |  |
| Signet ring cell | 30 | 18(60.0) | 18.7 |  |
| Others | 3 | 0(0.0) | - |  |
| Histological grade |  |  |  | 0.008 |
| Low grade | 54 | 23(42.6) | 59.3^a^ |  |
| High grade | 170 | 106(62.4) | 29.3 |  |
| Vascular invasion |  |  |  | <0.001 |
| Negative | 44 | 12(27.3) | 66.9^a^ |  |
| Positive | 180 | 117(65.0) | 27.4 |  |
| Neural invasion |  |  |  | 0.002 |
| Negative | 67 | 27(40.3) | 57.9^a^ |  |
| Positive | 157 | 102(65.0) | 29.3 |  |
| Postoperative chemotherapy |  |  |  | <0.001 |
| No | 125 | 82(65.6) | 22.6 |  |
| Yes | 101 | 49(48.5) | 79.0 |  |
| TNM stage |  |  |  | <0.001 |
| Ⅰ/Ⅱ | 66 | 19(28.8) | 66.9^a^ |  |
| Ⅲ | 158 | 110(69.6) | 23.1 |  |
| Molecular subgroups |  |  |  | 0.016 |
| MSI | 52 | 22(42.3) | 58.4^a^ |  |
| EBV^+^ | 11 | 5(45.5) | 49.9^a^ |  |
| EBV^−^/MSS | 161 | 102(63.4) | 27.4 |  |
| PD-L1 |  |  |  | 0.001 |
| Positive | 132 | 89(67.4) | 27.4 |  |
| Negative | 92 | 40(43.5) | 55.4^a^ |  |

MSI: Microsatellite instability; MSS: microsatellite stable; EBV: Epstein-Barr Virus; PD-L1: programmed cell death ligand 1; CTx: Chemotherapy.

^a^For these characteristics, less than half of patients were died, so mean overall survival (OS) time was presented when median OS could not be calculated.
