# Supplementary material for: Microsatellite instability and Epstein-Barr virus combined with PD-L1 could serve as a potential strategy for predicting the prognosis and efficacy of postoperative chemotherapy in gastric cancer
Source: PeerJ. 2021 May 18;9:e11481. doi: 10.7717/peerj.11481 (PMC8139270; doi:10.7717/peerj.11481)
Supplement: Supplemental Information 3 — MSS: microsatellite stable; EBV, Epstein-Barr Virus; PD-L1, programmed cell death ligand 1; CTx, Chemotherapy. HR: Hazard ratio; CI, Confidence interval. a95% CI was calculated with multivariate Cox regression, adjusting for the variables that P<0.10 from the univariate analysis, such as WHO classification, histological grade, vascular invasion, neural invasion, postoperative chemotherapy and TNM stage. [file peerj-09-11481-s003.docx]

**Table S3:**

**Multivariate analyses of risk factors affecting overall survival (OS) in 224 gastric cancer patients**

| Characteristics |  | HR | 95%CI | *P* |
| --- | --- | --- | --- | --- |
| Molecular subtypes | Non MSS/EBV^−^(n=63) | 1.00 |  | 0.031 |
|  | MSS/EBV^−^(n=161) | 1.610 | 1.046-2.479 |  |
| PD-L1 | Negative | 1.00 |  | 0.053 |
|  | Positive | 0.677 | 0.456-1.006 |  |
| TNM stage | Ⅰ/Ⅱ | 1.00 |  | 0.003 |
|  | Ⅲ | 2.274 | 1.330-3.887 |  |
| Vascular invasion | Negative | 1.00 |  | 0.007 |
|  | Positive | 2.436 | 1.271-4.671 |  |
| CTx | No | 1.00 |  | <0.001 |
|  | Yes | 0.473 | 0.329-0.681 |  |

MSS: microsatellite stable; EBV: Epstein-Barr Virus; PD-L1: programmed cell death ligand 1; CTx: Chemotherapy. HR: Hazard ratio; CI: Confidence interval.

^a^95%CI was calculated with multivariate Cox regression, adjusting for the variables that *P*<0.10 from the univariate analysis, such as WHO classification, histological grade, vascular invasion, neural invasion, postoperative chemotherapy and TNM stage.
