# Supplementary material for: Microsatellite instability and Epstein-Barr virus combined with PD-L1 could serve as a potential strategy for predicting the prognosis and efficacy of postoperative chemotherapy in gastric cancer
Source: PeerJ. 2021 May 18;9:e11481. doi: 10.7717/peerj.11481 (PMC8139270; doi:10.7717/peerj.11481)
Supplement: Supplemental Information 4 — MSS, microsatellite stable; EBV, Epstein-Barr Virus; PD-L1, programmed cell death ligand 1; CTx, Chemotherapy; HR, Hazard ratio; CI, Confidence interval. a95% CI was calculated with multivariate Cox regression, adjusting for the variables that P<0.10 from the univariate analysis, such as WHO classification, histological grade, vascular invasion, neural invasion, postoperative chemotherapy and TNM stage. [file peerj-09-11481-s004.docx]

**Table S4:**

**Multivariate analyses of risk factors affecting overall survival (OS) in MSS/EBV^−^ subgroup stratified for different PD-L1 expression (N=161)**

| MSS/EBV^−^ | Characteristics |  | HR (95%CI)^a^ | *P* |
| --- | --- | --- | --- | --- |
| PD-L1^−^(n=104) | CTx | No | 1.00 | <0.001 |
|  |  | Yes | 0.357(0.217-0.587) |  |
| PD-L1^+^(n=57) | CTx | No | 1.00 | 0.090 |
|  |  | Yes | 0.499(0.223-1.117) |  |

MSS: microsatellite stable; EBV: Epstein-Barr Virus; PD-L1: programmed cell death ligand 1; CTx: Chemotherapy. HR: Hazard ratio; CI: Confidence interval.

^a^95%CI was calculated with multivariate Cox regression, adjusting for the variables that *P*<0.10 from the univariate analysis, such as WHO classification, histological grade, vascular invasion, neural invasion, postoperative chemotherapy and TNM stage.
