# Supplementary figures and images for: Microsatellite instability and Epstein-Barr virus combined with PD-L1 could serve as a potential strategy for predicting the prognosis and efficacy of postoperative chemotherapy in gastric cancer
Source: PeerJ. 2021 May 18;9:e11481. doi: 10.7717/peerj.11481 (PMC8139270; doi:10.7717/peerj.11481)

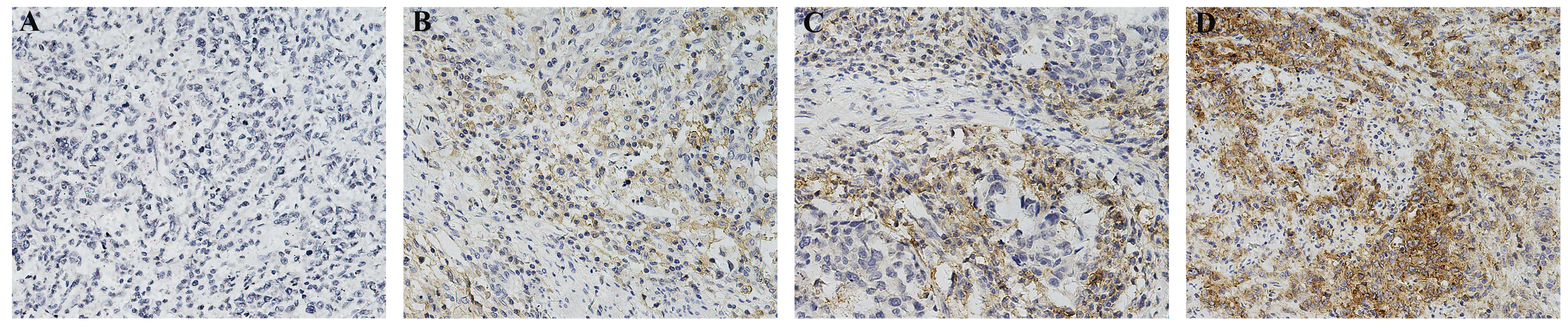

Supplement: Supplemental Information 5 — (A) Grade 0, no immunostaining. (B) Grade 1, weak staining. (C) Grade 2, moderate staining. (D) Grade 3, strong staining. [file peerj-09-11481-s005.png]

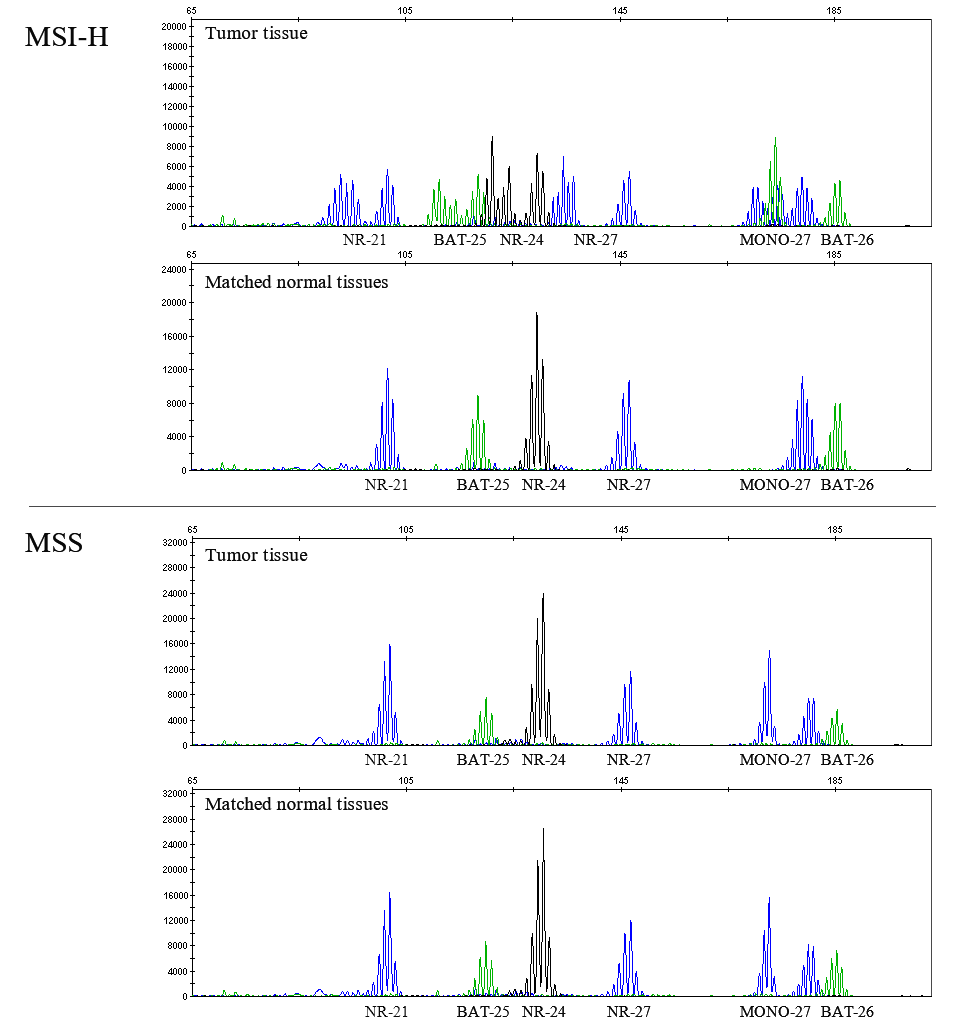

Supplement: Supplemental Information 6 — (A) An example of MSI-H case exhibiting alteration of allelic position of all six microsatellite markers. (B) A MSS case without instability at any microsatellite markers. [file peerj-09-11481-s006.png]

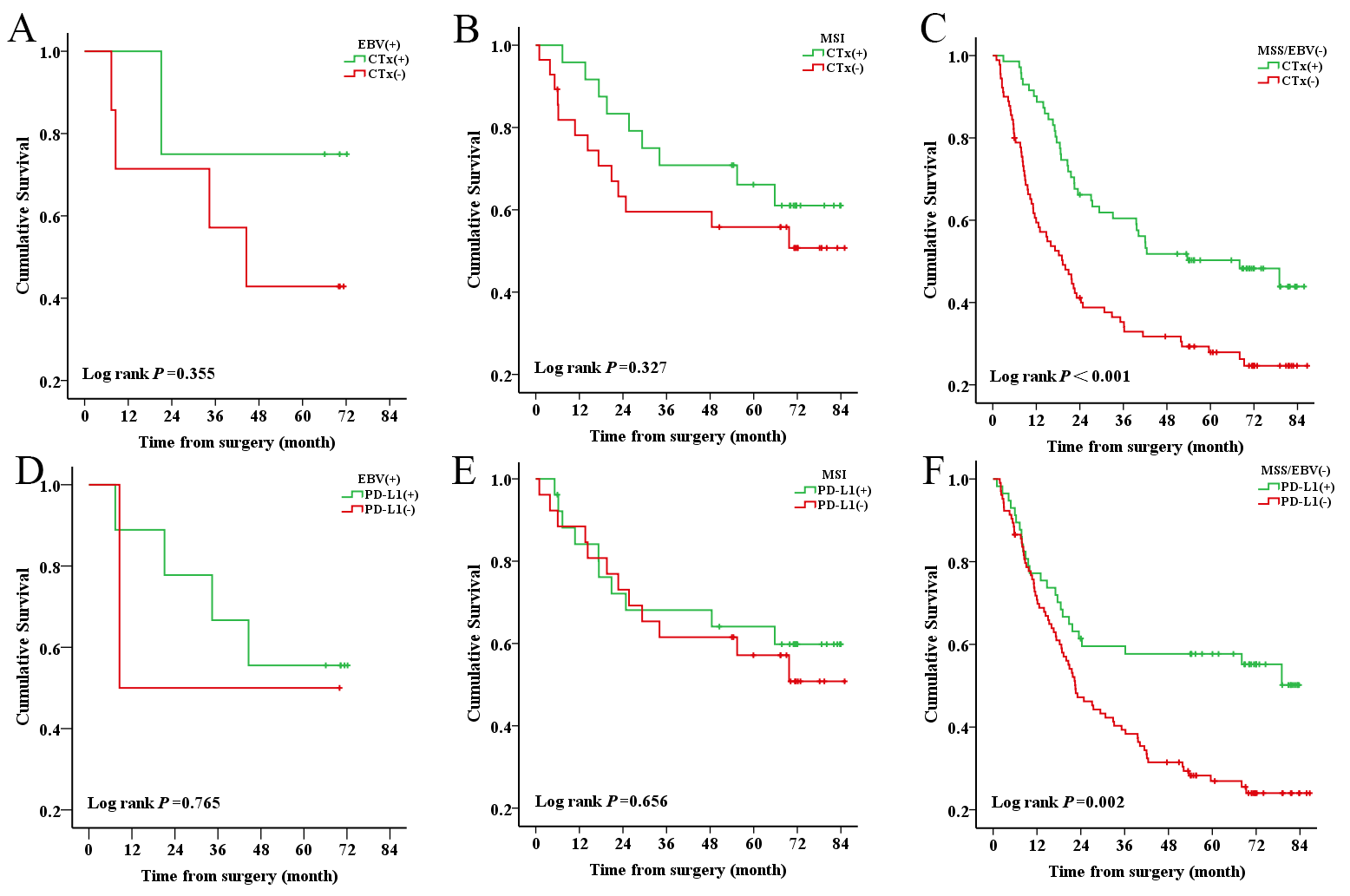

Supplement: Supplemental Information 7 — (A) CTx in EBV+ subgroup (B) CTx in MSI subgroup (C) CTx in MSS/EBV subgroup (D) PD-L1 expression in EBV+ subgroup (E) PD-L1 expression in MSI subgroup (F) PD-L1 expression in MSS/EBV subgroup. MSI, Microsatellite instability; MSS, Microsatellite stable; EBV, Epstein - Barr virus, PD-L1, Programmed cell death ligand 1; CTx, Chemotherapy. [file peerj-09-11481-s007.png]
